# Novel oleate hydratases and potential biotechnological applications

**DOI:** 10.1007/s00253-021-11465-x

**Published:** 2021-08-05

**Authors:** Peter Leon Hagedoorn, Frank Hollmann, Ulf Hanefeld

**Affiliations:** Department of Biotechnology, Delft University of Technology, Van der Maasweg 9, 2629 HZ Delft, The Netherlands

**Keywords:** Oleate hydratase, Protein engineering, 10-hydroxystearic acid, Biocatalysis

## Abstract

**Abstract:**

Oleate hydratase catalyses the addition of water to the CC double bond of oleic acid to produce (*R*)-10-hydroxystearic acid. The enzyme requires an FAD cofactor that functions to optimise the active site structure. A wide range of unsaturated fatty acids can be hydrated at the C10 and in some cases the C13 position. The substrate scope can be expanded using ‘decoy’ small carboxylic acids to convert small chain alkenes to secondary alcohols, albeit at low conversion rates. Systematic protein engineering and directed evolution to widen the substrate scope and increase the conversion rate is possible, supported by new high throughput screening assays that have been developed. Multi-enzyme cascades allow the formation of a wide range of products including keto-fatty acids, secondary alcohols, secondary amines and α,ω-dicarboxylic acids.

**Key points:**

• *Phylogenetically distinct oleate hydratases may exhibit mechanistic differences.*

• *Protein engineering to improve productivity and substrate scope is possible.*

• *Multi-enzymatic cascades greatly widen the product portfolio.*

## Introduction

**Scheme 1:**
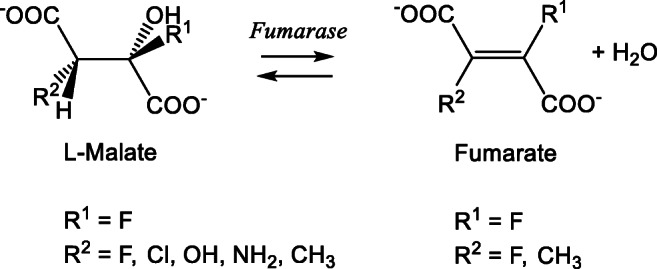
The substrate scope of fumarases

Hydratases or hydrolyases (EC 4.2.1) are enzymes that catalyse the addition of water to C=C double bonds. The BRENDA database contains approximately 200 different enzymes that are classified as hydrolyase constituting a highly diverse collection of hydratases and dehydratases that are structurally and mechanistically distinct. There are metal-free, flavin containing, iron-sulphur cluster containing and even molybdenum/tungsten cofactor dependent enzymes. This exemplifies that nature has found very diverse paths to catalyse this basic chemical reaction. The most notable hydratases from a biochemical point of view are the TCA enzymes aconitase and fumarase. Aconitase contains a catalytic (non-redox) iron-sulphur cluster and catalyses the isomerisation of citrate to isocitrate using a dehydration and hydrations step with cis-aconitate as intermediate product. Fumarase is a non-metallo enzyme in eukaryotes, although a structurally distinct iron-sulphur cluster containing fumarase is prominent in the bacterial world. Fumarase catalyses the reversible hydration of fumarate to L-malate (Scheme 1).

Most hydratases catalyse the conversion of a narrow substrate range with high efficiency. This is consistent with their general primary metabolic functions. For biotechnological applications, however, a broader substrate acceptance would be highly desired. And although chemical trickery has been shown to expand the substrate scope for some known hydratases, a general protein engineering strategy has not been successful to date. It is therefore highly interesting to search for novel hydratases using the ever expanding genomic databases. The structural diversity makes it difficult to discover truly novel hydratases. Several promising microbial activities for novel hydratases have been described in literature. These enzymes have proven to be hard to isolate and characterise (Busch et al. 2020a).

The exception to this picture is oleate hydratase. Oleate hydratase (Ohy) catalyses the selective addition of water to the C=C double bond in oleic acid (OA) to produce (*R*)-10-hydroxystearic acid (10-HSA). OA is the major fatty acid in olive and rapeseed oil and is thus an abundant, renewable resource. HSA has commercial applications as a plant-derived emollient, surfactant and thickener in cosmetics and as a monomer in the polymer industry. An example is 10-HSA as an active cosmetic ingredient which is marketed by DSM as Beauactive. Ohy was already discovered as a microbial activity in the 1960s (Davis et al. 1969; Wallen et al. 1962; Schütz et al. 2019). Although one apparently successful isolation of the enzyme was undertaken in 1991, it was only many years later in 2009 that the gene encoding this enzyme was discovered in *Elisabethkingia meningoseptica* (Bevers et al. 2009; Hou 1995). The coding sequence was already previously annotated as a myosin-cross-reactive antigen (MCRA), which is a curious name for a microbial enzyme, as myosin is one of the major proteins of muscles in humans and animals, and the myosin superfamily proteins only occur in Eukarya. Originally MCRA was discovered as a protein from the pathogenic bacterium *Streptococcus pyogenes* that upon infection can lead to the generation of antibodies that also react with human heart proteins causing acute rheumatic fever (Kil et al. 1994). It was later discovered that deletion of the Ohy/MCRA coding gene in *S. pyogenes* affected the virulence of this bacterium (Volkov et al. 2010). Interestingly, it was recently found that *Staphylococcus aureus* Ohy protects the pathogenic bacterium against the antimicrobial unsaturated fatty palmitoleic acid on human skin (Subramanian et al. 2019). So there is an interesting human medical relevance of Ohy. The old annotation of Ohy as MCRA serves as a warning that many misleading annotations occur in existing genomic and proteomic databases, and we should always be on the lookout for such errors. The misleading annotation as myosin-cross reactive-antigen persisted for many years after its original correction and can still be found in most prominent databases to date.

As a microbial activity oleate hydratase has already been reviewed long before the discovery and characterisation of the isolated enzyme (Hou 1995). More recently, a number of (mini-)reviews on hydratases, which include segments on fatty acid hydratases and oleate hydratases, have been published (Chen et al. 2015; Demming et al. 2018; Engleder and Pichler 2018; Resch and Hanefeld 2015). Several reviews on fatty acid hydratases, including oleate hydratases, have been published in the past few years. Zhang et al. provided an excellent review on the biotechnological potential of fatty acid hydratases, including the substrate scope and possibilities of protein engineering (Zhang et al. 2020b). Löwe and Gröger published a mini-review on the applications of fatty acid hydratases in organic synthesis, highlighting the possibility to produce industrially relevant chemical building blocks from renewable resources (Löwe and Gröger 2020).

Although all these reviews offer a comprehensive overview of the published literature and important biotechnological potential of fatty acid hydratases, these articles are superseded by new recent developments as new enzymes and structures have changed our understanding of the mechanism and opened new directions for biotechnological applications. This warrants an update on the reviews that have appeared so far. Here we attempt to provide the relevant consensus on the enzyme properties, including the limitations and issues with the reported data, the current understanding of the structure and mechanism of this enzyme, and the latest developments and outlooks on biocatalysis and biotechnological applications. As a point of focus, the conversion of oleic acid to 10-HSA by the fatty acid hydratase we call oleate hydratase is central in this review. It should be noted, however, that oleate hydratases have a wider substrate scope, overlapping with other fatty acid hydratases.

## Oleate hydratase

**Fig. 1:**
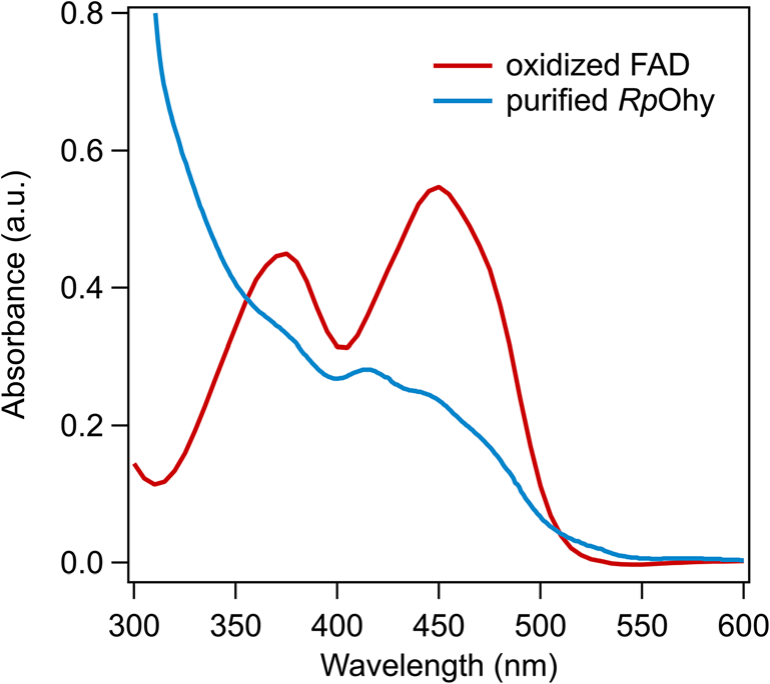
UV-visible spectrum of *Rhodococcus pyridinivorans* Ohy (Busch et al. 2020b)

Oleate hydratase (Ohy, EC 4.2.1.53) is an FAD-containing enzyme family that has some structural and functional variations. The enzyme from a range of bacteria has been isolated and characterised (Table 1). The FAD cofactor does not have a redox function as the redox state does not change during conversion (Engleder et al. 2015; Volkov et al. 2010). The reduced cofactor FADH_2_ is likely the relevant species under in vivo conditions (Engleder and Pichler 2018). Ohy containing FADH_2_ instead of FAD was found to be circa 10-fold more active in vitro (Engleder et al. 2015). The UV-vis properties several Ohys have been reported but the redox potentials for the cofactor have not been determined to date (Busch et al. 2020b; Engleder et al. 2015; Joo et al. 2012; Rosberg-Cody et al. 2011). The spectrum shows a somewhat unusual flavoprotein spectrum, which may reflect partially reduced cofactor (Fig. 1). Alternatively this spectrum could reflect oxidised FAD that is partially quenched due to protein binding. The current consensus is that the FAD cofactor has an essential structural role in the Ohy active site, which will be discussed in more detail below. It is not clear if and how the FAD is reduced in vivo. Fluorescence spectroscopy of Ohy under different conditions may resolve the ambiguous redox state of the FAD cofactor.

**Table 1:** Enzymatic properties oleate hydratases for which the isolated enzyme has been characterised

Organism	Name^a^	*k*_*cat*_ (s^−1^)	*K*_*M*_ (oleate) (μM)	Assay	Assay conditions	Reference
*Elizabethkingia meningoseptica*	*Em*Ohy	1.2 ± 0.2	110 ± 60	GC	50 mM HEPES pH 6, 2% v/v ethanol, 0.1-2 mM OA, 50 μg/ml enzyme, 150 rpm shaking, 25°C	(Engleder et al. 2015)
		0.17–0.48	100–600	GC	20 mM Tris pH 8.0, 50 mM NaCl, 2–8 mM substrate, 1000–2200 rpm shaking, 22–30°C	(Bevers et al. 2009)
*Lactobacillus acidophilus*	*La*Ohy1^b^	(4.3 ± 0.2)·10^−4^	28 ± 9	GC	100 mM KPi pH 6.0, 5% ethanol, 0.1 mM FAD, shaking, 37°C	(Eser et al. 2020)
	*La*Ohy2	0.11 ± 0.01	20 ± 9			
*Lysinibacillus fusiformis*	*Lf*Ohy	14.2 ± 0.1	540 ± 8	GC	50 mM Pipes pH 6.5, 4% ethanol, 0.2–20 mM substrate, 2–500 U/ml enzyme, 35°C	(Kim et al. 2012)
*Macrococcus caseolyticus*	*Mc*Ohy	7.83 ± 0.02	340 ± 2	GC	^c^50 mM Pipes pH 6.5, 2% (v/v) ethanol, 2 mM substrate, 0.01 mg/ml enzyme, 0.2 mM FAD, 25°C	(Joo et al. 2012)
*Paracoccus aminophilus*	*Pa*Ohy	1.01 ± 0.03	30 ± 4	GC	100 mM KPi pH 6.5, 0.01–1 mM, 1000 rpm shaking, 30°C	(Sun et al. 2021)
*Rhodococcus pyridinivorans*	*Rp*Ohy	19.8 ± 1.6	720 ± 70^d^	UV-vis coupled assay	50 mM PIPES pH 6.5, 10% (v/v) DMSO, 2 mM NAD^+^, 0.25–2.5 mM substrate, 0.3–1.5 μg/ml Ohy, 0.01–0.5 mg/ml ADH010, 25°C	(Busch et al. 2020b)
*Rhodococcus erythropolis*	*Re*Ohy	0.57 ± 0.08	490 ± 100	GC	20 mM Tris pH 7.2, 20 μM FAD, 0.09–1.44 mM substrate, 5 μM enzyme, 28°C	(Lorenzen et al. 2018)
*Staphylococcus aureus*	*Sa*Ohy	0.02^e^	2.1 ± 0.2^d^	TLC	50 mM KPi pH 6.0, 10 mM NaCl, 10 mM DTT, 50 μM FAD, 0.2 mg/ml BSA, 0–25 μM ^14^C OA, 0.05 mg/ml enzyme, 37°C	(Subramanian et al. 2019)
*Stenotrophomonas maltophilia*	*Sm*Ohy1	1.97	38.9	GC	50 mM citrate-phosphate buffer pH 6.0, 5% (v/v) DMSO, 0.004–1 mM substrate, 0.005–0.01 mg/ml enzyme, 35°C	(Kang et al. 2017a)
	*Sm*Ohy2	2.98	20.7			
*Stenotrophomonas nitritireducens*	*Sn*Ohy	78.2	21.5	GC	50 mM citrate-phosphate buffer pH 6.5, 5% (v/v) DMSO, 0.004–1 mM substrate, 0.05 mg/ml enzyme, 35°C	(Kang et al. 2017b)
*Streptococcus pyogenes M49*	*Sp*Ohy	1.12 ± 0.08	63 ± 6	GC	50 mM MES pH 6.1, 50 mM NaCl, 2% ethanol, 10% glycerol, 37°C	(Volkov et al. 2010)

Enzymes for which only conversions and data on cell extracts or whole cells were reported have been excluded. ^a^An attempt was made to use a consistent short name to designate the different enzymes, which is not always identical to the short name used in the literature, ^b^*L. acidophilus* Ohy1 has a strong preference for linoleic acid over oleic acid, ^c^preparation of oleic acid stock: 4 mM in 4% (v/v) ethanol, homogenised at 10000 rpm for 10 s. Substrate and enzyme were added in 1:1 (v:v) ratio. ^d^Cooperative kinetics was observed, so the *K*_*M*_ is a *K*_*0.5*_ with a Hill constant of 2.2–2.4, ^e^values estimated from the reported kinetic curves

Recombinantly expressed Ohy is often partially apo, without full incorporation of the FAD cofactor, which explains why the FAD cofactor was missed in the original publication describing the isolated protein (Bevers et al. 2009). Frequently FAD is added during enzyme assays to ensure full incorporation, and crystallisation conditions of *S. aureus Sa*Ohy required 0.75 mM FAD supplementation, even though the reported *K*_*M*_ for supplemented FAD for optimal activity is 2.1 μM (Subramanian et al. 2019). The *K*_*d*_ for FAD binding to *E. meningoseptica Em*Ohy was reported to be 1.8 μM measured by isothermal titration calorimetry (ITC) (Engleder et al. 2015). This suggests that FAD is not very tightly bound, at least in the recombinantly expressed enzymes.

Although a large number of different bacterial oleate hydratases have been recombinantly produced in *E. coli* and the biocatalytic conversion of different fatty acids has been shown, limited enzyme kinetic data and protein characterisation have been performed to date. For example of the Ohy’s from *Bifidobacterium breve*, *Bifidobacterium animalis*, *Lactobacillus acidophilus*, *Lactobacillus plantarum* and *Lactobacillus rhamnosus*, only the conversion of OA to 10-HSA of the isolated protein was shown, but no kinetic data or further characterisation has been reported (Rosberg-Cody et al. 2011; Yang et al. 2013). To make matters worse, in a number of cases, only the activity of cell lysates and whole cells and not data on isolated enzymes were reported: e.g. for Ohy from *Chryseobacterium gleum*, *Desulfobicrobium baculatum* and *Gemella morbillorum* (Schmid et al. 2016).

### Phylogeny

An extensive bioinformatic study to discover and analyse Ohy coding sequences using the BioCatNet database system resulted in the construction of the hydratase engineering database HyED (https://hyed.biocatnet.de/) which contains more than 2000 unique sequences (Schmid et al. 2016). The sequences were categorised on the basis of the amino acid sequence similarity in eleven distinct HFam homologous families. Each HFam family has at least 62% sequence identity among its members. Besides almost exclusively bacterial genes, only a relatively small number of fungal and archaeal sequences were found, but the enzymes encoded by these genes have not been successfully expressed to date. Among all Ohy’s, a conserved FAD binding site, at least in part, can be observed. Interestingly the catalytically important residue Glu122 is replaced by a Methionine in circa 30% of the sequences, mostly belonging to HFam 1, although HFam 3 family member *Rhodococcus erythropolis* Ohy *Re*Ohy contains the methionine instead of the conserved glutamate as well. The catalytically important Y241 (*Em*Ohy numbering) is conserved among all Ohy’s in the database.

### Enzyme activity assays

Measuring oleate hydratase activity is not trivial, as the solubility of the substrate and product are very low. OA can form micelles, vesicles and other structures in aqueous mixtures (Cistola et al. 1988; Dejanovic et al. 2011; Kaibara et al. 1997; Mele et al. 2018; Suga et al. 2016). The *pK*_*a*_ of the carboxylate group of oleic acid is strongly concentration dependent due to neighbouring effects (e.g. in vesicles) ranging from *pK*_*a*_ 5 to 10 (Kanicky and Shah 2002; Salentinig et al. 2010). At neutral pH, a small amount of sodium oleate will be formed, which can act as an emulsifier (Kaibara et al. 1997). Hence, it is possible that oleic acid/buffer emulsions may be formed. The kinetic parameters that have been previously reported for Ohy have not been corrected for the complex phase behaviour of the substrate OA. Therefore, these parameters (Table 1) should be used with care, as these values are without exception ‘apparent’ kinetic parameters, and are highly dependent on experimental conditions, such as the use of co-solvents. Although kinetic parameters of Ohy’s have been tabulated in several previous review articles, these values are not useful without considering the precise assay conditions.

A number of different activity assay methods have been developed. GC and GC-MS assays have been used in most reports (Table 1). Whole cell samples, cell extracts or isolated enzyme can be incubated with the substrate oleic acid in suspension with or without co-solvents. The product 10-HSA can be extracted using an organic solvent such as ethyl acetate. Subsequently the product is derivatised using silylation to make the product more volatile, e.g. by using *N,O*-bis(trimethylsilyl)trifluoroacetamide (BSTFA) as derivatising agent. The derivatised compounds can be separated using an apolar column, such as CP-Sil5 CB, and detected using an FID detector.

Fatty acid analysis using HPLC is well established (Tarola et al. 2012). It is therefore rather surprising that HPLC has been rarely used in literature to measure Ohy reactions. An HPLC method using extraction of fatty acids and derivatisation to phenacyl bromide fatty acid derivatives was successfully used to measure Ohy conversion of OA to 10-HSA in two species of ruminal bacteria *Selenomonas ruminantium* and *Enterococcus faecalis* (Hudson et al. 1995). Unreacted OA and produced 10-HSA were extracted and dried. Derivatisation was achieved by addition of 2-bromoacetophenone and trimethylamine in acetone and incubation at 100°C for 15 min. The derivatisation was stopped by the addition of acetic acid, and drying of the samples. The samples were analysed using HPLC with a C18 reversed phase column using UV detection. Recently, an HPLC-MS method to detect hydroxyl fatty acids, including 10-HSA, without a derivatisation step was reported, which could be interesting for measuring Ohy reactions (Kokotou et al. 2020).

The desire for an enzyme assay that can be used in high throughput to facilitate enzyme engineering led to the development of a screening method on the basis of a chemical follow-up reaction by forming chromogenic alkyl nitrites (Hiseni et al. 2014). This assay allowed the distinction between tertiary and primary/secondary alcohols, and was successful for the measurement of Ohy activity in a microtiter plate format, which in principle can be implemented in a high throughput screening platform.

**Scheme 2:**
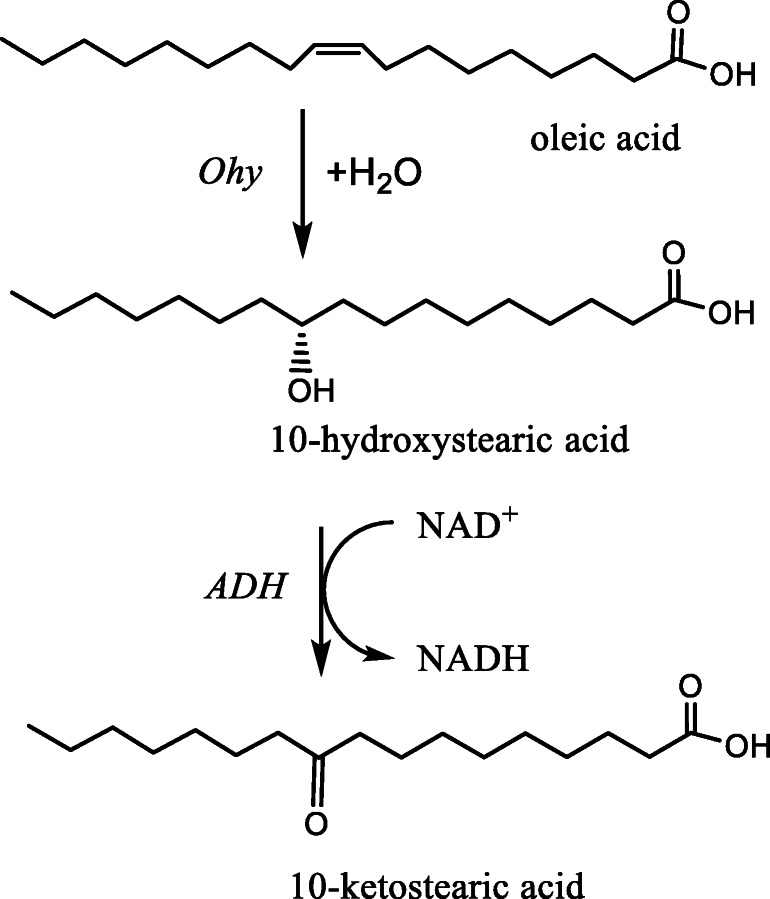
Coupled enzymatic UV-vis spectroscopic assay for Ohy activity

More recently a completely enzymatic coupled UV-vis spectrophotometric assay was developed that was adapted to a high throughput assay (Busch et al. 2020b; Sun et al. 2021). This assay is based on the coupling of the Ohy reaction with the subsequent enzymatic oxidation of 10-HSA to 10-ketostearic acid by an NAD^+^-dependent secondary alcohol dehydrogenase (Scheme 2). This allows the UV-vis spectrophotometric monitoring of NADH production over time as a measure of Ohy activity under the right conditions. The 10-HSA oxidation activity was already reported in microbial whole cell reactions (Niehaus et al. 1978), and a number of 10-HSA converting alcohol dehydrogenases have been identified (Huang et al. 2020; Wu et al. 2019). ADH010 and ADH020 out of a commercial screen of ten different NAD^+^-dependent alcohol dehydrogenases (Evoxx, Monheim am Rhein, Germany) were found to convert 10-HSA efficiently (Busch et al. 2020b). By coupling the reaction to the chemical oxidation of NADH by phenazine methosulfate (PMS) coupled to the reduction of MTT (3-(4,5-dimethylthiazol-2-yl)-2,5-diphenyltetrazolium bromide) forming its insoluble purple-coloured formazan, a feasible HTS assay was developed (Sun et al. 2021).

Interestingly cooperative kinetics was observed for *Rp*Ohy and *Sa*Ohy, which were the only two enzymes for which the activity was not determined using discontinuous GC assays (Table 1). It remains to be determined if these effects are due to the experimental conditions that were used or reflect true enzyme properties.

### Structure

**Fig. 2:**
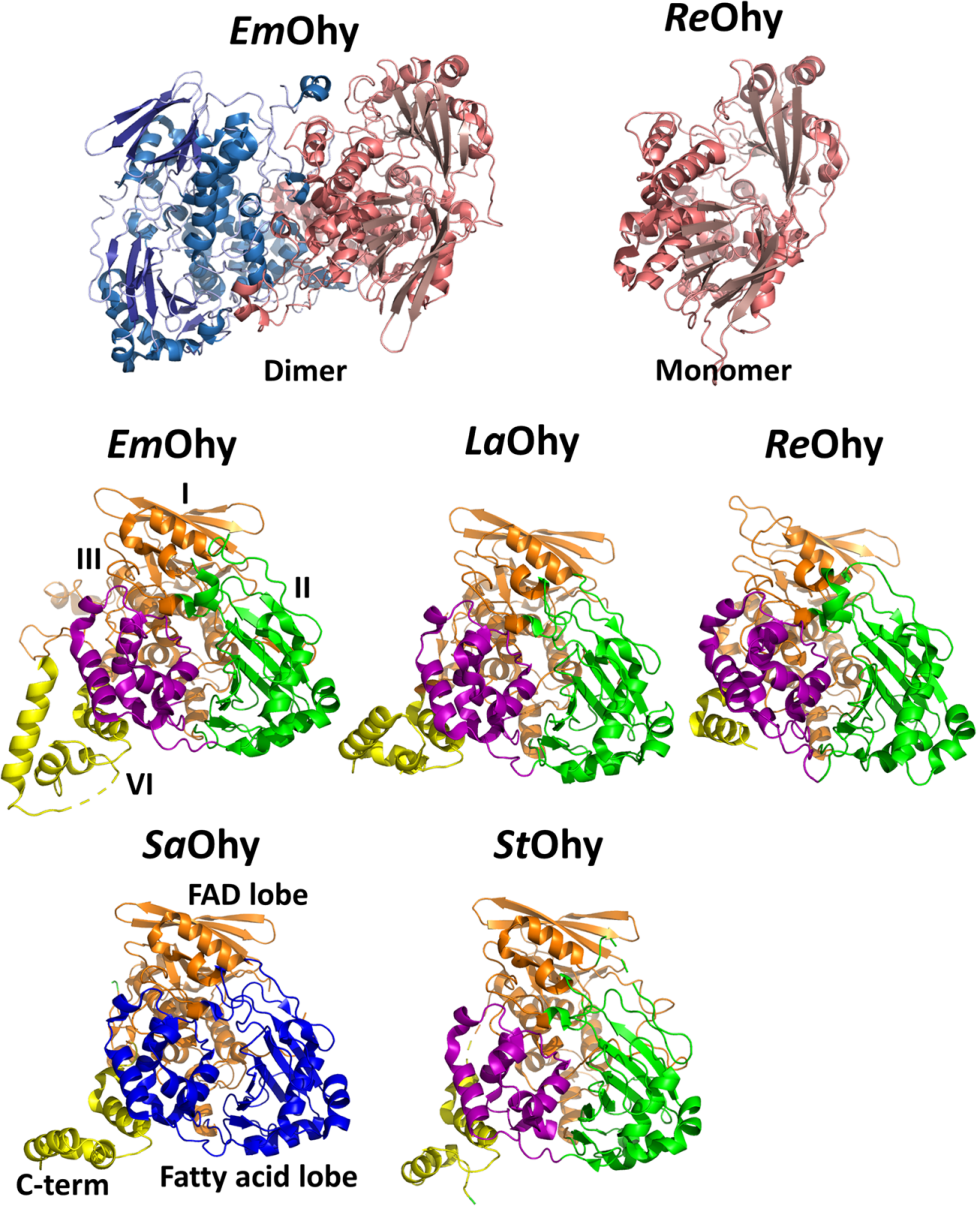
Overview of the structures of oleate hydratases showing the dimeric and monomer structures (top) and the domain organisation (bottom): domain I (orange), domain II (green), domain III (purple) and domain IV (yellow). For SaOhy, the domains are as follows: FAD lobe (orange), fatty acid lobe (blue) and C-terminal domain (yellow). The following pdb files were used: *Em*Ohy, 4uir; *La*Ohy, 4ia6; *Re*Ohy, 5odo; *Sa*Ohy, 7kaz; *St*Ohy, 5z70. The images were created using PyMOL

In 2013, the first structure of an oleate hydratase *L. acidophilus* Ohy (pdb entry 4ai6, Table 2) (Volkov et al. 2013) was reported. To date, 10 structures of 5 different oleate hydratases have been deposited (Fig. 2). All structures are homodimeric, except for *R. erythropolis Ohy* (pdb 5odo, Table 2). Only two structures contain the FAD cofactor, which is essential for activity, and several structures contain substrate or product.

**Table 2:** Structures of oleate hydratases

Organism	Name	Type	pdb entry	Remarks	Reference
*Elisabethkingia meningoseptica*	*Em*Ohy	HFam 11	4uir	FAD bound	(Engleder et al. 2015)
*Lactobacillus acidophilus*	*La*Ohy	HFam 2	4ia5	apo	(Volkov et al. 2013)
			4ia6	Substrate bound^a^	
*Rhodococcus erythropolis*	*Re*Ohy	HFam 3	5odo	apo	(Lorenzen et al. 2018)
*Staphylococcus aureus*	*Sa*Ohy	HFam 11	7kaz	E82A, substrate, product^b^ and FAD bound	(Radka et al. 2021)
			7kav	PEG bound	
			7kaw	PEG and FAD bound	
			7kax	E82A	
			7kay	E82A and substrate^c^ bound	
*Stenotrophomonas* sp. *KCTC 12332*	*St*Ohy	HFam 11	5z70	apo	(Park et al. 2018)

^a^Linoleic acid, ^b^10-HSA, ^c^OA

The structures of oleate hydratase are predominantly homodimeric, with each monomer consisting of four domains (Fig. 2). Three domains form together the FAD and substrate/product binding sites (domains I–III), and the fourth C-terminal domain (domain IV) provides a hydrophobic substrate access channel. There is structural evidence from *L. acidophilus* Ohy that the substrate bound conformation of one subunit influences the conformation of the other to facilitate substrate access, which could be consistent with the cooperative kinetics that has been observed in some Ohy’s (Volkov et al. 2013). The structure of *Em*Ohy showed a homodimer with one monomer FAD bound and one monomer apo (Engleder et al. 2015). The conserved active site residues Glu122 and Tyr241 (*Em*Ohy numbering) were found to be very important for the hydratase activity. Further evidence for conformational changes in the N-terminal loop region involved in the FAD binding part of the enzyme was provided by the *Stenotrophomonas* sp. KCTC12332 Ohy structure (Park et al. 2018). In the absence of bound FAD, this region is less structured and more flexible, and may serve as a ‘lid’ upon binding of the cofactor. The structure of *R. erythropolis* Ohy showed a monomeric protein, rather than the homodimer of all other reported structures (Lorenzen et al. 2018). The monomer has the same four domains as the other Ohy’s, although the C-terminal domain four is significantly shorter, lacking an alpha-helix involved in the dimerisation in the case of the other Ohy’s. This domain undergoes a large conformational change upon substrate binding. Interestingly the conserved glutamate residue of most Ohy’s was replaced by a methionine (M77 *Re*Ohy numbering). The M77E variant of *Re*Ohy showed a 5-fold reduction in activity compared to the WT enzyme, which indicates a distinct mechanism for this enzyme and possibly other HFam 3 type enzymes.

**Fig. 3:**
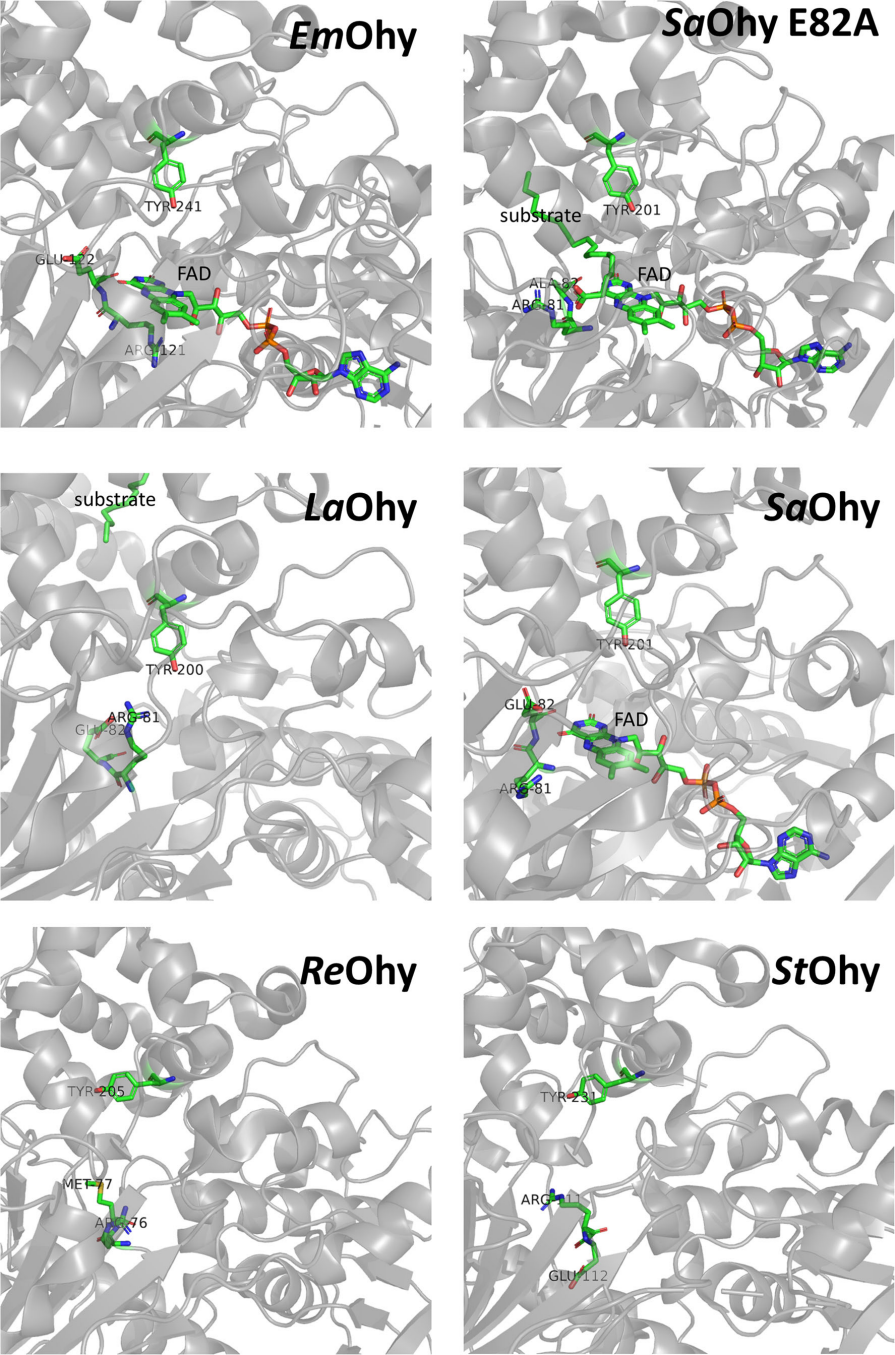
Active site structures of oleate hydratases with and without bound FAD and substrate. Important active site aminoacids are indicated. The following pdb files were used: *Em*Ohy, 4uir; *La*Ohy, 4ia6; *Re*Ohy, 5odo; *Sa*Ohy, 7kaw; *Sa*Ohy E82A, 7kaz; *St*Ohy, 5z70. The images were created using PyMOL

The most complete structures have been obtained for *S. aureus Sa*Ohy with OA and 10-HSA bound and with the FAD cofactor bound as well (Fig. 3) (Radka et al. 2021). Arg81 (*Sa*Ohy numbering) was found to serve as a ‘gatekeeper’ residue involved in the proper orientation of the fatty acid substrate. This structure led to an important revision of the proposed catalytic mechanism, which will be discussed below. Although the overall structure of the *Sa*Ohy is similar to the other Ohy’s, the structure was divided in three ‘functional’ domains rather than the four ‘structural’ domains (I to IV) that are described above. The three domains were named: FAD-lobe (domain I), fatty acid lobe (II and III) and C-terminal domain (IV) (Fig. 2).

### Catalytic mechanism

**Scheme 3:**
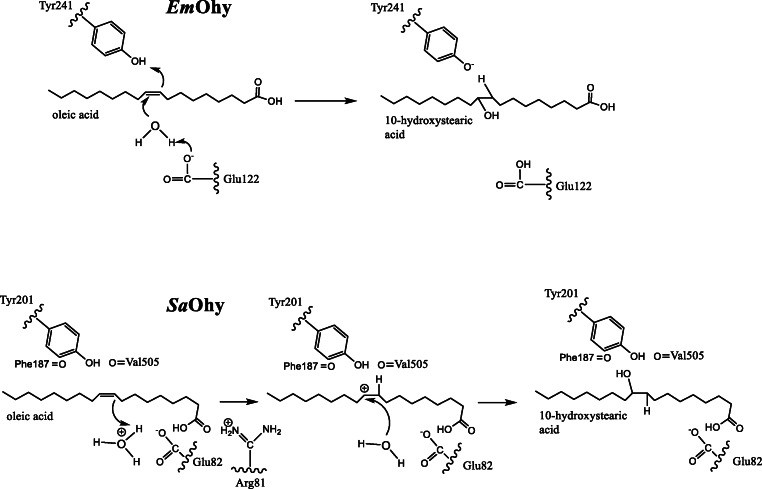
The reaction mechanisms that have been proposed for *Em*Ohy (top) and *Sa*Ohy (bottom)

Based on the structure of *Em*Ohy (Fig. 3), a catalytic mechanism has been proposed (Scheme 3) involving the protonation of the CC double bond by the conserved active site tyrosine (Y241 in *Em*Ohy, equivalent to Y201 in *Sa*Ohy), with subsequent nucleophilic attach of water, supported by deprotonation using the conserved glutamate (E122 in *Em*Ohy, equivalent to E82 in *Sa*Ohy) (Engleder et al. 2015). The function of the FAD cofactor, most likely FADH_2_, was proposed to be structural and to stabilise the transient positive charge on the substrate after the protonation step.

This mechanism has recently been superseded by a new one due to structural information of *Sa*Ohy (Fig. 3). A mechanism based on acid-base catalysis involving careful positioning of the substrate water and the fatty acids assisted by the FAD cofactor has been proposed (Radka et al. 2021). The conserved active site Glutamate (E122 in *Em*Ohy and E82 in *Sa*Ohy) is involved in stabilising the substrate water molecule as a hydronium ion (Scheme 3). The H^+^ from the hydronium ion attack the CC double bond producing a transient carbocation intermediate, which is subsequently attacked by the water to form the hydrated product. In this mechanism, FAD predominantly functions to expel most water molecules from the active site, and to properly orient the important residues Glu82 and Arg81 facilitating the proper order of the mechanistic steps. FAD does not form a stable prosthetic group for this enzyme but it released to facilitate product release. The potential role of the conserved tyrosine Y201 (Y241 in *Em*Ohy) in the catalytic mechanism as proposed by Engleder et al. is questionable as the tyrosyl oxygen forms a hydrogen bound with the backbone carbonyl of V505 in all structures of *Sau*Ohy, with or without substrate or product bound. This excludes the deprotonation of this tyrosine as the first step of the mechanism. The tyrosine is involved in a hydrogen bonding network that stabilised the bound product, and *Sau*Ohy Y201F was found to still stereoselectively add water to OA, albeit at a 10-fold reduction of the rate.

The mechanism of *Re*Ohy (HFam 3), which has a methionine (M77 *Re*Ohy numbering) instead of the conserved active site glutamate, remains to be resolved. Perhaps another active site residue can take over the role to activate the water molecule. This at least indicates that there may be different catalytic mechanisms for the oleate hydratases, which may hold for different HFam types.

### Substrate scope

**Scheme 4:**
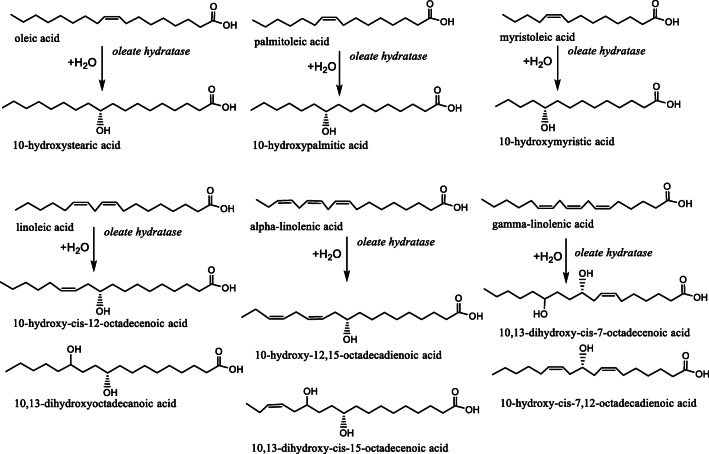
Substrate scope of oleate hydratases

Already for the original microbial activity of oleate hydratase (more generally fatty acid hydratase) and later for the isolated enzymes and expressed enzyme containing *E. coli* lysates, it was clearly shown that the enzyme is capable of hydrating a number of different ω-9 unsaturated fatty acids, including oleic acid (C18:1), palmitoleic acid (C16:1), myristoleic acid (C14:1) and linoleic acid (C18:2) (Scheme 4) (Hou 1995). In almost all cases, the product has the OH group on the C10 position, although for certain substrates, C13 has also been reported (Eser et al. 2020). Depending on the preferred substrate, some of the reported oleate hydratases may have to be renamed for example as a linoleic acid hydratase. It is clear that there is overlapping substrate specificity among fatty acid hydratases. A study of two distinct Ohys from *Rhodococcus* species showed that the enzymes have a complementary substrate scope, for which some substrates were only converted by *Re*Ohy and some only by *Rp*Ohy (Busch et al. 2020b). This confirms that protein engineering has potential to direct the substrate preference.

## Biotechnological applications of oleate hydratase

### Production of hydroxy fatty acids

Already since the discovery of the microbial Ohy activity in the 1960s, applications to produce hydroxyl fatty acids have been reported (Hou 1995; Koritala et al. 1989). Since the coding gene and structure are uncovered, tailored engineering has become feasible, which has increased the development of improved enzymes and approaches to enhance the substrate scope. Eser et al. showed that targeted mutations can influence the substrate specificity and regioselectivity of two related Ohy’s from *L. acidophilius* (Eser et al. 2020). The productivity of different enzyme systems for the hydration of different fatty acids has been reviewed excellently by Löwe and Gröger (Löwe and Gröger 2020). Space-time yields (STYs) for the production of 10-HSA ranging from 8 to 384 g L^−1^ h^−1^ have been reported. Bioprocess engineering of an *E. coli* strain overexpressing *St*Ohy resulted in a 10-HSA productivity of 46 g/L culture (Jeon et al. 2012).

The substrate scope of Ohy can be enhanced by using so-called decoy molecules. Hauer and co-workers used hexanoic acid as a short ‘decoy’ carboxylic acid, to allow the selective hydration of short chain alkenes by *Em*Ohy (Demming et al. 2019). Site-directed mutagenesis of a small hydrophobic amino acid A248 close to the active site improved the productivity. Though still in its infancy, this approach offers a very promising approach for the stereoselective hydration of a broader range of terminal and other non-activated C=C-double bonds. Hopefully, further improvements will turn this methodology truly practical for organic synthesis.

### Engineering Ohy for 10-HSA production

Rational mutagenesis of amino acids involved in the substrate binding site of *Em*Ohy resulted in variants with improved conversion in whole cell reactions after 96 h of OA derivatives in which the carboxylate group was replaced by different esters, alcohol, hydroxamic acid or amide groups (Engleder et al. 2019). For example, *Em*Ohy Q265A/T436A/N438A exhibited a 17.6-fold higher conversion rate for the propyl ester of OA compared to the WT enzyme, albeit with a low overall yield of 4%. This shows that the substrate scope of Ohy’s can be significantly enhanced using protein engineering.

Variants of *Paracoccus aminophilus* Ohy produced by directed evolution were found to have enhanced OA conversion activity and stability under particular reaction conditions (Sun et al. 2021). This was possible by the development of the enzymatic colorimetric high-throughput screening assay discussed above. Site-saturation mutagenesis of important active site residues resulted in only one improved variant. By combining different successful amino acid substitutions, the triple mutant F122L/F233L/T15N was obtained that exhibited a 4-fold higher *k*_*ca*t_, at a similar *K*_*M*_ compared to the WT enzyme. On the basis of a homology model of the structure of *Pa*Ohy, the molecular basis of the beneficial mutations was attributed to increasing hydrogen bonding interactions in different sites in the protein. Using the enzymatic cascade of Ohy and *Micrococcus luteus* secondary alcohol dehydrogenase, a STY of 540 g L^−1^ day^−1^ of 10-HSA and 10-ketostearic acid was obtained.

### Multi-step reactions to broaden the product scope

Hydroxylated fatty acids are interesting products with a range of applications as cosmetic ingredients or as antimicrobial active compounds. Beyond this, hydroxyl fatty acids also serve as building blocks for the synthesis of other chemical intermediates. For this, a range of cascade reactions involving further enzymatic conversion steps after the Ohy-catalysed double bond hydration have been designed. Especially Park and coworkers have pioneered a range of interesting cascades (Song et al. 2013; Song et al. 2014; Song et al. 2020). The first step in most of these cascades comprises the oxidation of the newly formed alcohol into the corresponding ketone as catalysed for example by the secondary alcohol dehydrogenase (ADH) from *Lactobacillus delbrueckii* or *M. luteus* (Seo et al. 2019b; Wu et al. 2019). Performing this reaction in a cell-free environment requires in situ regeneration of the oxidised nicotinamide cofactor, which can be achieved, e.g. by employing lactate dehydrogenase-catalysed reduction of pyruvic acid (NADH-dependent and NAD^+^-forming). Due to opposing pH optima of the hydration and the oxidation step, this reaction was performed in a one-pot two-step fashion including a pH switch between both reactions. Nevertheless, a STY of 217 g L^−1^ day^−1^ was obtained (using 5 g/L *Pa*Ohy and 0.25 g/L lyophylised cell extract of the other enzymes).

**Scheme 5:**
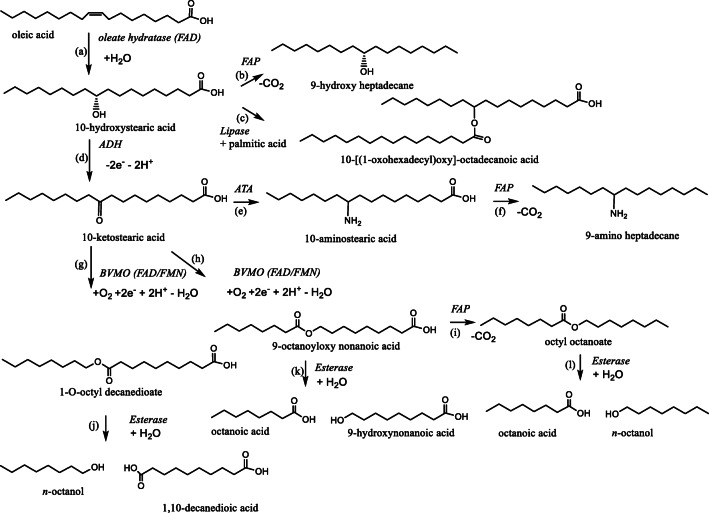
Overview of biocatalytic cascades with Ohy. FAP, fatty acid photodecarboxylase; ATA, amine transaminase; BVMO, Baeyer-Villiger monooxygenase. **a** Oleate hydratase-catalysed hydration of oleic acid to 10-hydroxystearic acid, **b** FAP-catalysed decarboxylation of 10-HSA to 9-hydroxy heptadecane, **c** lipase-catalysed esterification of 10-HSA to the FAHFA 10-[(1-oxohexadecyl)oxy]-octadecanoic acid, **d** secondary alcohol dehydrogenase-catalysed oxidation of 10-HSA to 10-ketostearic acid, **e** transamination catalysed by amine transaminase of 10-ketostearic acid to 10-aminostearic acid, **f** decarboxylation of 10-aminostearic acid to 9-amino heptadecane, **g** BVMO-catalysed oxidation of 10-ketostearic acid to 1-*O*-octyl decanedioate, **h** BVMO-catalysed oxidation of 10-ketostearic acid to 9-octanoyloxy nonanoic acid, **i** FAP-catalysed decarboxylation of 9-octanoyloxy nonanoic acid to octyl octanoate, **j** esterase-catalysed hydrolysis of 1-*O*-octyl decanedioate to *n*-octanol and 1,10-decanedioic acid, **k** esterase-catalysed hydrolysis of 9-octanoyloxy nonanoic acid to octanoic acid and 9-hydroxynonanoic acid, and **l** esterase-catalysed hydrolysis of octyl octanoate to octanoic acid and *n*-octanol

The resulting keto fatty acids can be further transformed further into useful products (Scheme 5). For example, esters can be obtained using Baeyer-Villiger oxidases (BVMOs) (Song et al. 2013). Depending on the selectivity of the BVMO used, the O atom can be inserted either on the carboxy terminal substituent of the keto group or on its alkyl terminal side. As a result, after hydrolase-catalysed cleavage of the esters, either dicarboxylic acids or ω-hydroxy carboxylic acids (together with the primary alcohols formed during the hydrolysis) can be obtained (Seo et al. 2018; Song et al. 2014). Both products are interesting, bio-based building blocks for polyesters. Another possibility of further valorising the Ohy-ADH-derived fatty acid ketones is to perform a transaminase-catalysed reductive aminations yielding fatty amines. By engineering the NAD^+^-dependent ADH from *M. luteus* to a more effective NADP^+^-dependent enzyme, a redox neutral bi-enzymatic cascade with the NADPH-dependent BVMO was obtained (Seo et al. 2019b).

Co-expression of the Ohy, ADH and BVMO in *E.coli* resulted in an effective whole cell biocatalyst containing the whole cascade (Seo et al. 2019a). By optimising the protein expression and by using an engineered BVMO, a yield of *n*-nonanoic acid and 9-hydroxynonnoic acid of 6 mmol/g dry cells was obtained. Another effective strategy based on the same cascade was to combine different whole cell biocatalysts and cell free enzymes in one pot for the production of C11 nylon monomers (undecanedioic acid and 11-aminoundecanoic acid) from ricinoleic acid (12-hydroxy-9-*cis*-octadecenoic acid) (Kim et al. 2020). The key for the effective biotransformation was to use adsorbent polymeric beads (Sepabeads S825) to bind the hydroxyl fatty acid and products, which ameliorated their enzyme inhibitory effects.

More recently, the aforementioned cascades have been extended by use of a new, photoactivated fatty acid decarboxylase from *Chlorella variabilis* (*Cv*FAP) (Sorigué et al. 2017). Combining both enzymes enabled the transformation of a range of unsaturated fatty acids into secondary alcohols (Zhang et al. 2020a). This cascade had to be performed in a one-pot two-step fashion in order to avoid the *Cv*FAP-catalysed decarboxylation of the unsaturated fatty acid starting materials. *Cv*FAP can also be used to decarboxylate the products obtained through the LdADH/BVMO or LdADH/TA cascades (Scheme 5) (Cha et al. 2020).

Another expansion of the scope of Ohy in biotransformations is the production of fatty acid esters of hydroxyl fatty acids (FAHFAs). FAHFAs are bioactive compounds with therapeutic potential, e.g. for the treatment of diabetes. Guo and co-workers were able to create a bi-enzymatic cascade containing *La*Ohy and *Candida Antarctica* lipase A (CalA) to generate different FAHFAs (Zhang et al. 2021). Using the *La*Ohy and CalA in a one-pot biphasic system with oleic acid and palmitic acid as substrate, the palmitate ester of 10-HSA (10-[(1-oxohexadecyl)oxy]-octadecanoic acid) was obtained with 48% conversion and 25% isolated yield.

### Immobilisation of Ohy

The immobilisation of enzymes is performed to improve their stability, to allow for reaction engineering (organic solvents or continuous reactions) and recuperation and reuse (Hanefeld 2013; Hanefeld et al. 2009). With these targets in mind, oleate hydratase was immobilised onto different carrier materials covalently as well as via non-covalent approaches, in the presence or absence of additives. The additives lead to initially higher activity; however, this might also be ascribed to the altered reaction conditions in their presence as discussed above. All immobilisation procedures lead to a significant loss of activity. This might be due to the fact that in all procedures involved several washing steps, leading to a loss of the essential FAD. Adsorption on Celite 545, ionic interactions with chitosan and coordination via the his tag yielded disappointing results, as did straightforward cross-linking to form a cross-linked enzyme aggregate (CLEA). Sol-gel entrapments with many variations resulted in complete deactivation. However, covalent attachment onto magnetic chitosan composite macro-particles was successful with a recovery of activity of 24% and enhanced enzyme stability. The easy to handle particles could be recycled 5 times with minor loss of activity in batch reactions (Todea et al. 2015).
